# Efficient Driving of Piezoelectric Transducers Using a Biaxial Driving Technique

**DOI:** 10.1371/journal.pone.0139178

**Published:** 2015-09-29

**Authors:** Samuel Pichardo, Rafael R. C. Silva, Oleg Rubel, Laura Curiel

**Affiliations:** 1 Thunder Bay Regional Research Institute, Thunder Bay, Ontario, Canada; 2 Electrical Engineering, Lakehead University, Thunder Bay, Ontario, Canada; 3 Materials Science and Engineering, McMaster University, Hamilton, Ontario, Canada; University of Nebraska-Lincoln, UNITED STATES

## Abstract

Efficient driving of piezoelectric materials is desirable when operating transducers for biomedical applications such as high intensity focused ultrasound (HIFU) or ultrasound imaging. More efficient operation reduces the electric power required to produce the desired bioeffect or contrast. Our preliminary work [Cole *et al*. Journal of Physics: Condensed Matter. 2014;26(13):135901.] suggested that driving transducers by applying orthogonal electric fields can significantly reduce the coercivity that opposes ferroelectric switching. We present here the experimental validation of this biaxial driving technique using piezoelectric ceramics typically used in HIFU. A set of narrow-band transducers was fabricated with two sets of electrodes placed in an orthogonal configuration (following the propagation and the lateral mode). The geometry of the ceramic was chosen to have a resonance frequency similar for the propagation and the lateral mode. The average (± s.d.) resonance frequency of the samples was 465.1 (± 1.5) kHz. Experiments were conducted in which each pair of electrodes was driven independently and measurements of effective acoustic power were obtained using the radiation force method. The efficiency (acoustic/electric power) of the biaxial driving method was compared to the results obtained when driving the ceramic using electrodes placed only in the pole direction. Our results indicate that the biaxial method increases efficiency from 50% to 125% relative to the using a single electric field.

## Introduction

In biomedical applications of ultrasound, the efficiency of a piezoelectric actuator is often defined as the ratio between the acoustic power obtained over the applied electrical power. More efficient driving of piezoelectric materials is desirable when designing and building transducers for biomedical applications such as high intensity focused ultrasound (HIFU), ultrasound imaging and ultrasonic motors. More efficient operation reduces the electric power needed to produce the desired bioeffect or contrast in imaging. For applications requiring continuous operation at high power (thousands of Joules), such as HIFU, more efficient energy conversion translates into less internal heat generation and consequently reduces the constrains for cooling, which is often needed in the design of actuators. Piezoelectric actuators are commonly driven by applying the electric field along the poling axis in order to maximize their mechanical response. To better understand the piezoelectricity phenomenon, and evaluate potential solutions to increase efficiency, our group has performed preliminary microscopic theoretical studies using modern polarization theory to establish a relation between atomic structure and dielectric dissipation of ferroelectric materials [1–3]. In this preliminary work, we adapted Density Functional Theory [4, 5] to better understand the energetics of ferroelectric switching driven by an external electric field. This modelling is based on first principle calculations and differs from many of the traditional macroscopic models of piezoelectric response [6–9].

**Fig 1:**
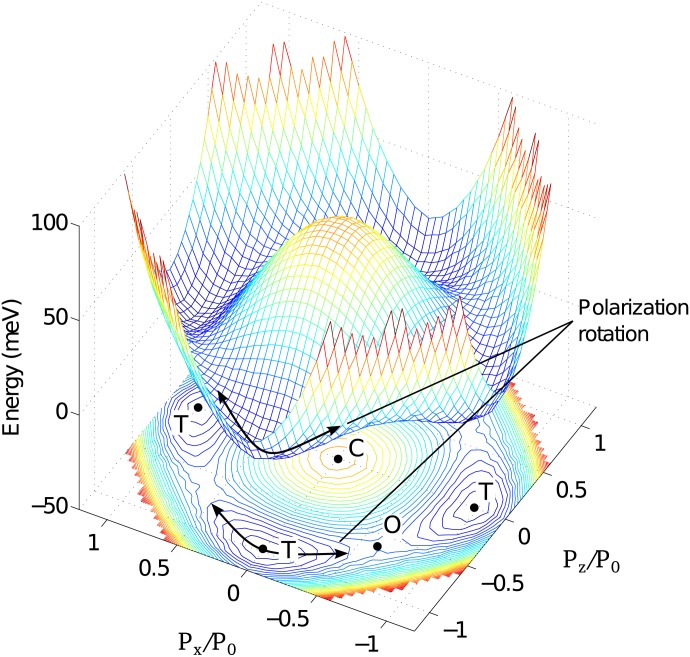
Free energy surface for polarization evolution in the (010) plane of PbTiO3 [3]. The labels C, T and O refer to cubic, tetragonal and orthorhombic structures, respectively. The arrows indicate evolution of polarization in a rotational manner.

The free energy profile for single-domain ferroelectric PbTiO_3_ obtained from first principle calculations is shown in Fig 1. The high energy barrier for cubic structure and a much lower barrier for orthorhombic structure below the Curie temperature create favourable conditions for the polarization rotation [10–12]. Fu and Cohen [13] have shown that a polarization rotation can enhance the piezoelectric response. This rotation mechanism was proposed in Ref. [13] to explain the “giant” piezoelectric response in PZT-PT and PMT-NT materials, where a non-aligned field alternates the material strain vectors between tetragonal and rhombohedral configurations. During the structural transformation associated with polarization rotation, the polarization vector **P** does not vanish, but changes its direction while maintaining a magnitude almost identical to spontaneous polarization *P*_0_ (Fig 1). These arguments suggest that the uniaxial electric field along the polling direction may not be the optimal driving method for guiding polarization rotation along a curved path. The proposed method of electrical excitation used in the present study allows to exploit polarization rotation to further enhance the mechanical response of actuators.

A possible strategy to facilitate polarization rotation along a curved path uses two-dimensional excitation with active modulation of the *E*_*x*_(*t*) and *E*_*z*_(*t*) components of the applied field. Our work in [3] suggested that the application of two orthogonal electric fields, instead of one as commonly done in most applications, can significantly reduce the coercivity that opposes the ferroelectric switching. A specific condition was predicted whereby a rotating electric field would result in a reduction in the coercivity. This rotation can be achieved by dephasing two sinusoidal electric fields. Theoretical studies based on the Landau-Ginzburg-Devonshire phenomenological model showed a similar reduction in the coercive field when a supplemental external mechanical stress is applied to the piezoelectric material. When this external stress is applied in the polarization direction the coercive field is reduced and the piezoelectric response is enhanced [14]. In our previous numerical work, we obtained an enhanced response by modifying the pattern of the applied electric field rather than a supplemental mechanical stress. Other experimental work have also obtained a similar enhancement by using a direct voltage field to produce a supplemental stress and used the effect to achieve higher pressure output for lithotripsy pulses [15].

The goal of the present study is to validate the predictions of our theoretical work on the reduction of coercivity when driving piezoelectric actuators. For this purpose, a set of narrow-band transducers were fabricated with two sets of electrodes placed in an orthogonal configuration. Transducers were cut to have resonance frequencies as similar as possible in both orthogonal directions. A series of experiments were then conducted where each pair of electrodes was driven independently and measurements of effective acoustic power were obtained using a radiation force method.

## Materials and Methods

### Sample preparations

**Fig 2:**
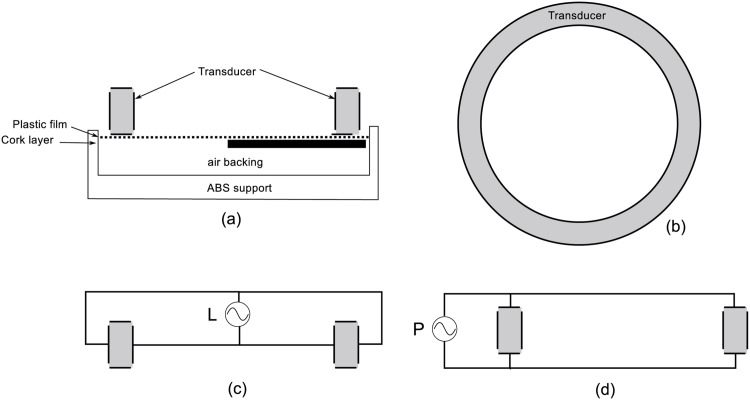
Schema of transducer preparation and driving. (a) Cross-section view of the ring-shaped transducer. (b) Top view of the ring-shaped transducer and absorber. (c) Connection of “lateral” (L) mode electrodes where the power supply drives the inner and outer faces of the ring. (d) Connection of “propagation” (P) mode electrodes where the power supply drives the top and bottom faces of the ring.

Three (3) transducer samples were characterized. Each sample was made of modified Lead zirconate titanate (PZT) composite typically used for HIFU devices (DL47, Del Piezo Specialities, West Palm Beach, FL). All samples have a ring geometry as shown in Fig 2.

The outer diameter of the transducer was 12 mm; with a ring width of 3 mm and a height of 6 mm. In addition, the natural resonance frequency of the transducers was specified to be as close as possible to 500 kHz. The transducers were configured as air-backed, using a cork layer below the bottom face of the ring (Fig 2a). A 0.05mm-thick plastic film was used to isolate the cork from the transducer. The ensemble was secured on a 3D-printed ABS support (Makerbot, Brooklyn, NY, USA) using epoxy glue (301, epoxy technology, Billerica, MA).

As shown in Fig 2c and 2d, two pairs of electrodes were placed following the propagation (P) mode and the lateral (L) mode. Each pair was driven independently by its own power supply. The P mode electrodes were placed on the top and bottom of the ring and the L mode electrodes on the outer and inner walls of the ring. The transducers were poled along the propagation direction P. Each mode was electrically characterized independently using a network analyzer (8751A, Hewlett Packard, Kobe Instruments Division, Hyogo, Japan) and matching circuits were built using solenoids and capacitors to adapt each mode to 50 Ω.

### Measurements of acoustic power vs. phase

**Fig 3:**
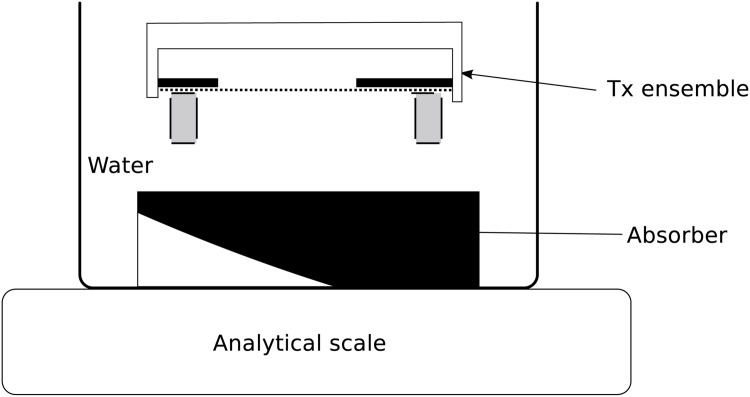
Radiation force setup.

As shown in Fig 3, each transducer was characterized using a radiation force setup [16]. The principle of this setup assumes that all acoustic energy generated by the transducer is absorbed, resulting in mechanical displacement that can be measured as a change of mass on a scale. A 6-cm diameter cylindrical absorber made specifically for radiation force measurements (HAM A, Precision Acoustics, Dorchester, Dorset, UK) was placed at the bottom of a water container, which sat on top of the plate of an analytical scale (PI-225D, Denver Instruments, Bohemia, NY, USA). The transducer was placed 2 cm above the absorber and the container was filled with deionized and degassed water (less than 1 ppm of oxygen).

The P and L mode electrodes were driven using a dual-channel function generator (33522A, Agilent Technologies, Santa Clara, CA) programmed in continuous mode. Signals were amplified using linear amplifiers (A150, ENI, Rochester, NY). The signal driving the L-electrodes was programmed with a phase shift *ϕ* relative to P-electrodes.

The acoustic power as a function of the relative phase *W*_*A*_(*ϕ*) was calculated using [16]

$$\begin{array}{c} W_{A}\left(\phi \right)=m\left(\phi \right)gc, \end{array}$$ (1)

where *m* is the mass (kg) measured using an analytic scale when the transducer is excited, *g* is the gravity constant (9.81 m⋅s^−2^) and *c* is the speed of sound in water (1481 m⋅s^−1^) at room temperature. The efficiency of the transducer *η*(*ϕ*) was calculated with [16]

$$\begin{array}{c} \eta \left(\phi \right)=\frac{W_{A}\left(\phi \right)}{W_{E}\left(\phi \right)}, \end{array}$$ (2)

where *W*_*E*_(*ϕ*) = *W*_*EP*_(*ϕ*) + *W*_*EL*_(*ϕ*) is the effective electrical power (forward minus reflected) on the P (*W*_*EP*_) and L (*W*_*EL*_) electrodes. *W*_*EP*_ and *W*_*EP*_ were measured simultaneously using power meters (N1914A, Agilent Technologies, Santa Clara, CA) and −30 dB couplers (C5085-10, Werlatone, Patterson, New York). Power was configured individually to deliver 1 effective electrical W in continuous mode in each *W*_*EP*_ and *W*_*EL*_ for a total of 2 electrical W. To measure the gain in efficiency of the P+L configuration, a series of acquisitions was performed driving only the P electrodes calibrated with *W*_*EP*_ equal to 2 W. Using this configuration, the P+L driving mode can be compared to the P mode alone under the same initial electrical power conditions.

For each transducer, three (3) identical series of measurements were performed where *ϕ* was changed from 0 to 360° in steps of 5°. In each series a total of 72 radiation force acquisitions were performed and the order of the phase values was randomized. Because the dimensions of the container were small (total water volume of 180 cm^3^), evaporation effects were present and compensated. Each acquisition started only after the scale detected stable readings using its internal filter set to “normal” mode. Scale measurements were also done without ultrasound every 0.5 s during 10 s and a linear fitting of the weight loss over time caused by evaporation was calculated. This fitting was then used to correct *m*(*ϕ*) at the time of transducer excitation. Since weight losses due to evaporation follow a linear relationship, the *R* ^2^ value of the fitting was also used to determine the stability of the scale measurements. Only measurements of evaporation showing a linear fitting with *R* ^2^ equal or larger to 0.9 were kept for analysis. After the evaporation measurements, the transducer was excited for 8 s and the scale reading was taken immediately prior to the end of the excitation. The value of *m*(*ϕ*) was corrected with the linear fitting of evaporation and then used to calculate *W*_*A*_ using Eq (1). The acquisition was controlled with a laptop computer (Lattitude E6500, Core 2 P8600 at 2.4 GHz, 4 GB RAM, Dell Computers, Round Rock, TX) running Matlab R2009 (Mathworks, Natick, MA).

## Results

Table 1 shows the electrical characterization of the transducers. The resonance frequencies of the P and L electrodes were in general very similar showing a global average (± s.d.) of 465.1(±1.5) kHz. Because the resonance frequency of both modes for each transducer was not exactly the same, experiments were conducted under the following conditions:

1. Driving both the P and L electrodes using their average frequency, which was calculated on a per transducer basis.
2. Driving both the P and L electrodes using the P-resonance frequency.
3. Driving both the P and L electrodes using the L-resonance frequency.
4. Driving each of the P and L electrodes using their individual resonance frequency.

Conditions 1 to 3 used the same frequency for both sets of electrodes while condition 4 used a different frequency for each set.

**Table 1:** Electrical characterization of transducers.

Tx #	mode	Impedance before matching (Ω)	Impedance after matching (Ω)	Resonance frequency (kHz)	Average (kHz)	Difference (kHz)
1	P	250 − *j*270	49 − *j*2	463.7	464	0.65
	L	127 − *j*44	50 − *j*0.3	464.35		
2	P	226 − *j*158	48 − *j*1	465.65	467.7	2.1
	L	104 − *j*42	49 − *j*0.1	467.8		
3	P	290 − *j*230	49 − *j*1.6	466	464.6	2.8
	L	117 − *j*56	50 − *j*1	463.15		

### P and L electrodes driven at the same frequency

**Fig 4:**
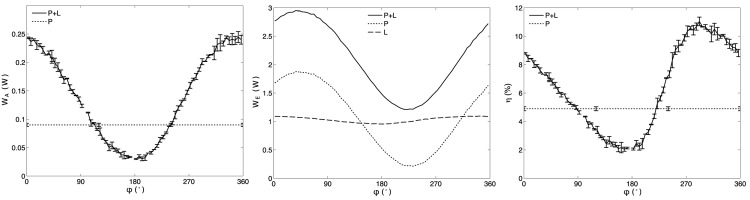
Plots of *W*_*A*_(*ϕ*), *W*_*E*_(*ϕ*) and *η*(*ϕ*) for a transducer sample when driving the P and L electrodes with the same average frequency and initially using 1 effective electric W in each set of electrodes. On the plots for *W*_*A*_(*ϕ*) and *η*(*ϕ*), the results for the P+L driving technique are compared to the case where only the P electrodes are driven using 2 effective electric W. The error bars indicate the standard deviation between the 3 repetitions of experiments at the same value of *ϕ*. The plot of *W*_*E*_(*ϕ*) shows the individual effective electrical power on each of the P (*W*_*EP*_) and L (*W*_*EL*_) electrodes and their sum P+L (*W*_*E*_) when driving the P and L electrodes simultaneously. When only the P electrodes were driven (not shown), the values of *W*_*EP*_ and *W*_*EL*_ were 2 W and 0 W, respectively.

Fig 4 shows plots of *W*_*A*_, *W*_*E*_, and *η* for one of the transducer samples when driving both the P and L electrodes with the same average frequency. The values of *W*_*A*_ and *η* are also plotted when driving only the P electrodes. Similar results were obtained when driving both the P and L electrodes either with the P-resonance or L-resonance frequency. Plots indicate a sinusoidal-shaped relationship between the acoustic power *W*_*A*_ and the phase *ϕ* applied on the *L* electrodes. *W*_*A*_ almost tripled from 0.09 W, when using only the P-electrodes, to 0.24 W when using the P+L mode and a phase *ϕ* of 352°. However, when *ϕ* was set at 182° *W*_*A*_ decreased to 0.03 W, which was a third of the baseline value. This result indicates that *ϕ* needs to be carefully selected to ensure an enhancement in the output acoustic power. It is worth noting that the measured effective electrical power *W*_*E*_ also showed a sinusoidal-type function for *ϕ*, suggesting that the electrical response of the transducer in each channel changes when applying the two electric fields simultaneously. This effect was more pronounced for the P electrode which resulted in *W*_*EP*_ ranging from 0.22 W with *ϕ* = 227° to 1.8 W with *ϕ* = 47°. For the L electrode, *W*_*EL*_ changed from 0.95 W with *ϕ* = 177° to 1.09 W with *ϕ* = 347°. For both sets of electrodes the difference in *ϕ* between the maximum and minimum was close to 180°. There was also a difference of 55° between the phases where *W*_*E*_ and *W*_*A*_ show their maximum. Our results indicated that the efficiency *η* could be doubled from 4.9% when only driving the P electrodes, to 11.4% when driving the P+L electrodes at a phase *ϕ* of 292°.

**Fig 5:**
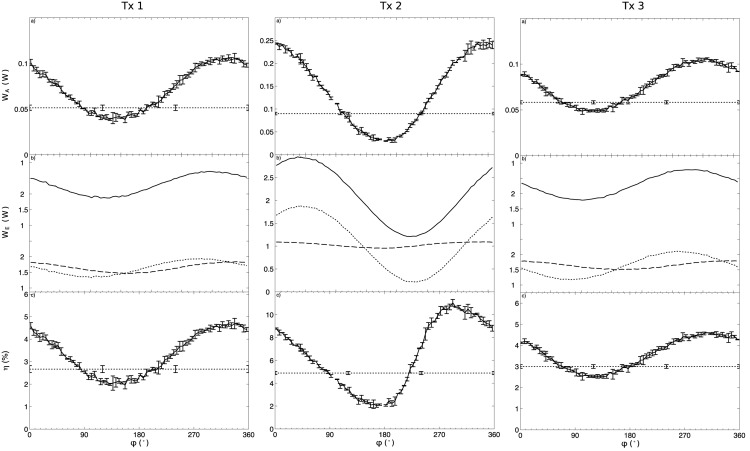
Plots of *W*_*A*_ (a), *W*_*E*_ (b) and *η* (c) as a function of *ϕ* for all transducers (Tx.#) when driving both the P and L electrodes at the same average frequency. On the plots for *W*_*A*_(*ϕ*) and *η*(*ϕ*), the results for the “P+L” (−) driving technique are compared to the case when driving only the “P” (⋅⋅⋅) electrodes. The error bars indicate the standard deviation between 3 repetitions of the experiments at the same value of *ϕ*. The plot of *W*_*E*_(*ϕ*) shows the individual effective electrical power on each of the electrodes: P (*W*_*EP*_, ⋅⋅⋅), L (*W*_*EL*_,—--) and their sum P+L (*W*_*E*_, −) when driving both P and L simultaneously. When only the P electrodes were driven (not shown), values of *W*_*EP*_ and *W*_*EL*_ were 2 W and 0 W, respectively.

Fig 5 shows the ensemble of results (*W*_*A*_, *W*_*E*_, *η*) for all transducers when their P and L electrodes were driven at their average frequency. The transducers all showed similar results producing higher efficiency when the transducers were driven simultaneously with the P+L driving method than when driving them with the P electrodes only. The maximal gain in efficiency observed for transducers #1, #2 and #3 was, respectively, +74%, 124% and +53%. In each of the 3 transducers, *η* was maximal for a value of *ϕ* where *W*_*EP*_ and *W*_*EL*_ were both close to 1 W, as they were individually configured prior to the experiment.

Table 2 shows the summary of the results for the observed maximal and minimal efficiency when driving the transducers with the dual-mode technique under the four testing conditions indicated above. Table 3 shows a similar summary for the maximal and minimal acoustic power. The results in both tables indicate that the optimal phase was frequency dependant, and that the optimal value of *ϕ* translated into values of *W*_*EP*_ and *W*_*EP*_ close to 1 W, as they were individually configured. For each combination, when driving transducers at same frequency, transducers #1 and #3 showed both maximal *η* and *W*_*A*_ at same value of *ϕ*. For transducer #2, the peak of *η* was found 60° off the peak observed for *W*_*A*_ when driving electrodes at their average frequency. This difference in *ϕ* was reduced to 10° when driving both sets of electrodes at the P-mode resonance frequency, and reduced to to 20° when using the L-mode resonance frequency.

**Table 2:** Summary of results of maximal and minimal *η* found for each transducer. “n.t.” stands for non-observable trend to locate minimum or maximum values.

			Baseline	Maximum					Minimum				
Tx #	Freq P	Freq L	*η*	*η*	*ϕ*	*W*_*A*_	*W*_*EP*_	*W*_*EL*_	*η*	*ϕ*	*W*_*A*_	*W*_*EP*_	*W*_*EL*_
1	464	464	2.67	4.70	327	0.11	1.14	1.13	1.95	157	0.04	0.99	0.98
	463.7	463.7	2.62	5.87	307	0.12	1.03	1.07	1.42	152	0.03	0.90	0.98
	464.35	464.35	2.94	5.73	292	0.12	1.03	1.07	1.64	157	0.03	0.91	0.97
	463.7	464.35	2.69	3.35	n.t.	0.07	1.06	1.06	3.35	n.t.	0.07	1.06	1.06
2	467.7	467.7	4.89	11.04	292	0.20	0.75	1.07	2.04	167	0.03	0.69	0.96
	465.65	465.65	4.53	7.50	322	0.16	1.01	1.08	1.72	147	0.03	0.89	0.93
	467.8	467.8	4.68	8.21	332	0.17	0.99	1.09	1.82	137	0.03	0.87	0.94
	465.65	467.8	4.83	6.35	n.t.	0.13	1.07	1.03	6.35	n.t.	0.13	1.07	1.03
3	464.6	464.6	3.00	4.60	307	0.11	1.19	1.11	2.52	132	0.05	0.93	1.02
	466	466	3.44	5.16	307	0.12	1.23	1.05	2.84	112	0.05	0.79	0.98
	463.15	463.15	3.05	4.65	317	0.11	1.21	1.07	2.45	152	0.04	0.87	0.96
	466	463.15	3.23	3.09	n.t.	0.07	1.05	1.06	3.09	n.t.	0.07	1.05	1.06

**Table 3:** Summary of results of maximal and minimal *W*_*A*_ found for each transducer. “n.t” stands for non observable trend to locate minimum or maximum values.

			Baseline	Maximum					Minimum				
Tx #	Freq P	Freq L	*W*_*A*_	*W*_*A*_	*ϕ*	*η*	*W*_*EP*_	*W*_*EL*_	*W*_*A*_	*ϕ*	*η*	*W*_*EP*_	*W*_*EL*_
1	464.35	464.35	0.05	0.11	327	0.11	1.14	1.13	0.04	157	0.04	0.99	0.98
	463.7	463.7	0.05	0.12	307	0.12	1.03	1.07	0.03	152	0.03	0.90	0.98
	464.35	464.35	0.06	0.12	292	5.73	1.03	1.07	0.03	157	1.64	0.91	0.97
	463.7	464.35	0.05	0.07	n.t.	3.35	1.06	1.06	0.07	n.t.	3.35	1.06	1.06
2	467.7	467.7	0.09	0.24	352	9.17	1.57	1.09	0.03	182	2.06	0.50	0.96
	465.65	465.65	0.08	0.16	312	7.48	1.02	1.08	0.03	147	1.72	0.89	0.93
	467.8	467.8	0.08	0.17	312	8.19	1.03	1.08	0.03	137	1.82	0.87	0.94
	465.65	467.8	0.09	0.13	n.t.	6.35	1.07	1.03	0.13	n.t.	6.35	1.07	1.03
3	464.6	464.6	0.06	0.11	307	4.60	1.19	1.11	0.05	102	2.53	0.88	1.02
	466	466	0.07	0.12	307	5.16	1.23	1.05	0.05	112	2.84	0.79	0.98
	463.15	463.15	0.06	0.11	317	4.65	1.21	1.07	0.04	132	2.46	0.83	0.96
	466	463.15	0.06	0.07	n.t.	3.09	1.05	1.06	0.07	n.t.	3.09	1.05	1.06

### P and L electrodes each driven at its resonance frequency

**Fig 6:**
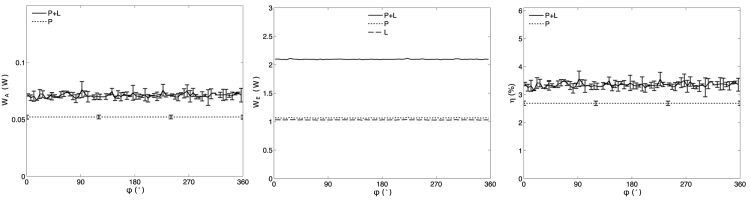
Plots of *W*_*A*_(*ϕ*), *W*_*E*_(*ϕ*) and *η*(*ϕ*) for transducer #2 when driving each of the P and L electrodes at their individual resonance frequency and initially using 1 effective electric W in each set of electrodes. On the plots for *W*_*A*_(*ϕ*) and *η*(*ϕ*), results of the P+L driving technique are compared to the case where only the P electrodes are driven using 2 effective electric W. The error bars indicate standard deviation between 3 repetitions of the experiments at the same value of *ϕ*. The plot of *W*_*E*_(*ϕ*) shows the individual effective electrical power on each of the P (*W*_*EP*_) and L (*W*_*EL*_) electrodes and their sum P+L (*W*_*E*_) when driving both P and L simultaneously. When only the P electrodes were driven (not shown), the values of *W*_*EP*_ and *W*_*EL*_ were 2 W and 0 W, respectively.

**Fig 7:**
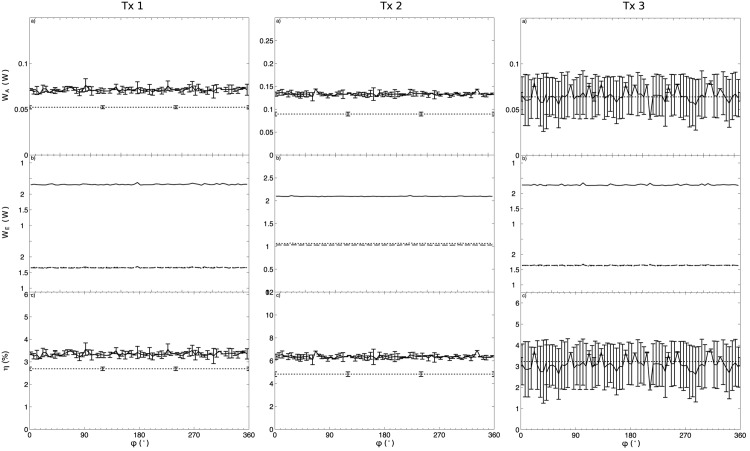
Plots of *W*_*A*_ (a), *W*_*E*_ (b) and *η* (c) as a function of *ϕ* for all transducers (Tx.#) when driving each of the P and L electrodes at their individual resonance frequency. The plots of *W*_*A*_(*ϕ*) (a) and *η*(*ϕ*) (c) show the results for the “P+L” (−) driving technique compared to the “P” (⋅⋅⋅) electrodes only. The error bars indicate the standard deviation between 3 repetitions of the experiments for the same value of *ϕ*. The plot of *W*_*E*_(*ϕ*) (b) shows the individual effective electrical power for each of the electrodes P (*W*_*EP*_, ⋅⋅⋅), L (*W*_*EL*_,—--) and their sum P+L (*W*_*E*_, −) when driving both P and L electrodes simultaneously. When only the P electrodes were driven (not shown), the values of *W*_*EP*_ and *W*_*EL*_ were 2 W and 0 W, respectively.

Fig 6 shows plots of *W*_*A*_, *W*_*E*_, and *η* for a transducer sample where each of its P and L electrodes were driven at their individual resonance frequency. In contrast to the results seen when driving P and L electrode at the same frequency, no observable trend as a function of *ϕ* could be observed. Fig 7 show the ensemble of results of *W*_*A*_, *W*_*E*_ and *η* for all the transducers. A constant increase in *η* was observed for transducers #1 and #2 when both electrodes were driven, but again no trend was observed and this gain was inferior to driving both electrodes at the same frequency. A summary of the results observed when driving electrodes P and L at their individual resonance frequency is included in Tables 2 and 3.

## Discussion

The results presented in this study indicate that a more efficient conversion of electrical power to forward acoustic power can be achieved in piezoelectric materials by applying two orthogonal electric fields. These measurements are in agreement with our previous numerical studies that predicted this phenomenon. Furthermore, the predictions in [3] indicated that the coercive field is highly anisotropic, and it was anticipated that the ferroelectric hysteresis would be sensitive to the direction of the applied electric field. The first principles modelling used in [1–3] differs from most macroscopic phenomenological modelling since the total energy losses in the former model are linked to the coercive field that opposes the external electric field. For the macriscopic phenomenological approach, the mechanical losses and the electric losses are often approximated separately and have been of great value in characterizing the losses of resonant or non-resonant driving of piezoelectric actuators [7, 9]. In first principle modelling materials are modelled as a perfect quasi-infinite layer; this type of approach is not suited to model differences between resonant and off-resonant driving techniques since this difference depends on macroscopic properties such as the actuator thickness. However, the first principle approach has brought some new insight into the energy losses that take place at the atomic level, opening new opportunities such as the biaxial driving method proposed here.

The choice of the ring configuration for this test was primarily for simplicity reasons in order to fabricate a simple device, easy to mount and operate in the frequency range of therapeutic ultrasound (around 500 kHz). Our setup for radiation force assumed plane wave conditions typically used in flat circular sources, which are not necessarily applicable for the ring configuration used in this experiment. The measurements obtained by the scale are thus a sum of the wave coming from the top face of the transducer and a partial contribution from the inner face of the ring transducer. Effects of clamping were potentially present but, as noted in Tables 2 and 3, those effects were uniform across all samples.

Our study was limited to a set frequency and operational mode, and many conditions remain to be explored. For example, it may be worth driving transducers at higher harmonics and in combinations of modes, such as driving the P-mode at the fundamental frequency and the L-mode at the 3rd harmonic. It is also worth exploring the potential effects of driving the transducers under broadband conditions as it is done in imaging applications. The proposed method requires two independent power lines per transducer, which doubles the complexity to drive transducer arrays. However, given an optimal fixed value for the phase, it is possible to split one common signal to produce the required delay using a filter circuit in series with the L-electrodes.

The proposed technique requires that both orthogonal dimensions be tailored to the central frequency of operation. In the case of the tested material, this requirement translated into a width of the ceramic that was half its height. However, using higher harmonics on the L-mode could facilitate fabrication of larger ceramics; a 3-times larger ceramic on the L dimension should in principle resonate at its 3rd harmonic, at the same frequency as the fundamental frequency of the P-mode. The requirement for application of orthogonal electric fields limits the possibility of using ceramic shapes that do not allow the placement of two set of orthogonal electrodes, such as a circular piston. Nevertheless, the proposed configuration of electrodes is well suited to a ring geometry, as used in this study, or prismatic shapes, which are ideal for linear arrays or even 2D-arrays.

## Conclusions

A new technique for driving piezoelectric materials was presented with the purpose of increasing operation efficiency under narrow band conditions. The technique consists of applying two spatially orthogonal electric fields. In this study, three samples of ring-shaped piezoelectric ceramics were fabricated and driven using the technique. Ring-shaped piezoelectric ceramics were poled from top to bottom and the electrodes were placed on all four sides of the ring. Both the height and thickness of the ring transducer were optimized to obtain similar resonance frequencies in both dimensions. The average (± s.d.) resonance frequency among all samples was 465.1(±1.5) kHz. A radiation force system was used to calculate the efficiency (conversion from electrical power to acoustic power) of the new driving method and was compared to driving the ceramics using electrodes placed solely in the pole direction. Our results indicate that the biaxial method increases efficiency, depending on the sample, from 50% to 125% when compared to applying a single electric field in the direction of the pole.
